# Enhancing the antifungal and antioxidant activity of *Agaricus bisporus* extract using gamma radiation: implications for shelf-life extension of fresh-cut apples

**DOI:** 10.1007/s10123-026-00806-3

**Published:** 2026-05-02

**Authors:** Mohamed S. Abd El-Al, Doaa A. Emam, Eman Araby, Abeer A. Khattab

**Affiliations:** 1 Radiation Microbiology Department, National Centre for Radiation Research and Technology (NCRRT), Egyptian Atomic Energy Authority, Cairo, Egypt; 2 Botany and Microbiology Department, Faculty of Science, Benha University, Benha, Egypt

**Keywords:** *Agaricus bisporus*, Antifungal activity, Gamma radiation, GC–MS profiling, Fruit preservation, Enzymatic browning, Microbial quality

## Abstract

Fresh-cut fruits are highly perishable due to enzymatic browning and microbial spoilage following tissue disruption. This study investigated whether γ-irradiation of the edible mushroom *Agaricus bisporus* enhances its antioxidant and antifungal properties and whether the treated extract can be applied as a natural preservative for fresh-cut apples. Ethanolic extracts of *A. bisporus* were irradiated at 0–8.0 kGy and evaluated against six apple-spoiling fungi (*Penicillium expansum*, *P. chrysogenum*, *Botrytis cinerea*, *Fusarium oxysporum*, *Aspergillus flavus*, and *A. niger*). Among the tested solvents, ethanol yielded the most potent extract, showing a 14-mm inhibition zone against *P. chrysogenum*. Irradiation at 6.0 kGy significantly increased total phenolic content (3.25 mg GAE g⁻¹) and DPPH scavenging activity (46.3% at 100 µg mL⁻¹) (*p* < 0.05). The 6.0 kGy extract inhibited fungal enzymes—protease (40%), cellulase (35%), and lipase (45%)—and caused hyphal deformation, as observed by SEM. Sorbitol protection assays indicated cell-wall targeting. GC–MS profiling revealed irradiation-induced modifications in the fatty acid and sterol compositions. When applied at 10% (w/v) to fresh-cut apples stored at 4 °C for 10 days, the irradiated extract reduced browning, retained firmness, and decreased fungal and bacterial counts by 1.3 and 1.6 log CFU g⁻¹, respectively. Overall, mild γ-irradiation (6.0 kGy) enhanced the bioactivity and preservative potential of *A. bisporus* extract, supporting its application as a natural, clean-label agent for fresh-cut produce preservation.

**Supplementary Information:**

The online version contains supplementary material available at https://doi.org/10.1007/s10123-026-00806-3.

## Introduction

The preservation of fresh-cut fruits is a significant challenge in the food industry, as they are highly susceptible to microbial spoilage, enzymatic browning, loss of firmness, and oxidative degradation. Fresh-cut fruit remains in high demand because it is easy to prepare and aligns with changing lifestyles, as more people seek ready-to-eat and ready-to-use products. These spoilage issues are especially prominent in climacteric fruits, which are metabolically active and spoil much faster than non-climacteric fruits. Apple (*Malus domestica*) is particularly vulnerable to post-cutting deterioration caused by polyphenol oxidase–mediated browning and fungal colonization. Various species from different genera, such as *Penicillium*, *Botrytis*, *Alternaria*, *Fusarium*, *Monilinia*, and *Aspergillus*, contribute to apple spoilage. However, two fungi—*Penicillium expansum* and *Botrytis cinerea*—are responsible for the most significant losses. These fungi not only diminish sensory quality but may also produce mycotoxins like patulin, raising food safety concerns. Postharvest losses result from microbial deterioration of apples; reports indicate that worldwide losses could reach 25% of total apple production (Khadiri et al. 2024).

Extending the storage life of fresh-cut apples while maintaining their quality has become a pressing issue, necessitating innovative approaches that are both effective and safe for consumers (Allegra et al. 2024). Conventional methods of delaying degradation, such as chemical antibrowning agents and synthetic fungicides, are becoming increasingly limited due to regulatory constraints and customer demand for “clean-label” alternatives (Khadiri et al. 2024). Polyphenol oxidase inhibitors are used to regulate browning enzymatically using chemical methods in two ways: by dipping in aqueous solutions enhanced with synthetic additives or by integrating edible coating formulations with active packaging (Fan 2023). For example, *Galla rhois* extract inhibited apple juice browning for over two weeks *via* gallotannin derivatives, showing potential as a natural additive (Lee et al. 2022). In this context, natural bioactive compounds have gained considerable attention as alternatives to synthetic preservatives. Various natural plant extracts have already been tested as anti-browning agents in freshly cut apple slices (Zhang et al. 2022). Plant-derived phenolics and essential oils have shown promise, although many of these methods face challenges such as sensory acceptability, durability, and limited antifungal activity (Soetjipto and Aminu 2022).

Identifying novel sources of natural antimicrobials that are both effective and compatible with fresh-cut fruit is therefore a priority. On the other hand, bioactive compounds from edible fungi have garnered interest recently due to their antimicrobial and antioxidant properties (Abdelshafy et al. 2022). For example, Koc et al. (2020) demonstrated the antioxidative and antibacterial properties of chitosan-fungal extracts. Edible mushrooms, particularly *A. bisporus* (white button mushroom), are a rich source of phenolic compounds, sterols, and other metabolites with antioxidant and antimicrobial properties. Several studies have demonstrated that mushroom extracts can inhibit bacterial and fungal growth; however, their potency varies depending on the extraction method and chemical availability (Abdelshafy et al. 2022; Yadav and Negi, 2021). White button mushrooms are also rich in phenolic compounds and have considerable antioxidant activity (Dhawan and Chakraborty 2024). For instance, Khan et al. (2016) reported that *A. bisporus* extract has the highest inhibitory activity of diphenyl-2-picrylhydrazyl (87.88 ± 5.6%). Several antimicrobial peptides have been found in mushrooms; the majority of them are endogenous peptides that may be classified into three types: antifungal, antibacterial, and antiviral peptides (Moussa 2022; Riaz et al. 2022).

*γ*-irradiation has emerged as a promising tool to modulate the chemical profile of plant and fungal matrices. At moderate doses, irradiation can disrupt cell walls and liberate bound phenolics, enhancing antioxidant potential, while also generating novel compounds that may contribute to antimicrobial activity (Hwang et al., 2021; Song et al. 2022). Building on this rationale, the present study aimed to investigate whether *γ*-irradiation of *A. bisporus* enhances the antifungal and antioxidant activities and evaluates the application of these extracts for maintaining quality and reducing microbial spoilage in fresh-cut apples during refrigerated storage.

## Materials and methods

### Chemicals and media

All chemicals, solvents, and media used in this study were of analytical grade. Potato dextrose agar (PDA), Plate Count Agar (PCA), and casein were obtained from HiMedia (Mumbai, India). Tween 80, methanol, ethanol, and ethyl acetate were purchased from Piochem Laboratory Chemicals (Cairo, Egypt). Folin–Ciocalteu reagent, diphenyl-2-picryl-hydrazyl (DPPH), tert-butylhydroquinone (TBHQ), sorbitol, sodium carbonate, carboxymethylcellulose, p-nitrophenyl palmitate, and gallic acid were purchased from Sigma-Aldrich (St. Louis, MO, USA). Saline solution (0.85% NaCl) was prepared using sodium chloride obtained from El-Gomhoria Co. (Cairo, Egypt). Fluconazole and Amphotericin B were purchased from Merck (Darmstadt, Germany). The apple treatment experiments additionally used citric acid and ascorbic acid (Sigma-Aldrich, St. Louis, MO, USA), and distilled water.

### Preparation of mushroom samples and irradiation

Fresh white button mushrooms (*Agaricus bisporus*) were obtained from the Agricultural Research Centre (Giza, Egypt; harvested March 2024). Mushrooms were rinsed in sterile distilled water, dried at 45 °C for 72 h, and ground into a fine powder (particle size ~ 2 μm) using a sterile mortar. The resulting powder was stored at 4 °C for no longer than 24 h before irradiation. The ground mushroom was packaged in polyethylene pouches (20 g each) and irradiated at the National Center for Radiation Research and Technology (NCRRT, Cairo, Egypt). Samples were exposed to doses of 0 (control), 2.0, 4.0, 6.0, and 8.0 kGy at a dose rate of 0.7 kGy/h under ambient conditions (25 °C). Three independent batches were prepared for all assays. The dose rate was verified using an alanine dosimeter system (Bruker Instruments, Rheinstetten, Germany). After irradiation, samples were sealed and stored at 4 °C in sterile containers for further use.

### Fungal strains and culture conditions

Postharvest fungal pathogens used in this study (*Penicillium expansum*, *P. chrysogenum*, *Botrytis cinerea*, *Fusarium oxysporum*, *Aspergillus flavus*, and *A. niger*) were obtained from the well-maintained culture collection of the food microbiology laboratory, NCRRT, Cairo, Egypt. Each strain has been assigned a unique code for reference: MS-PE-01 (*P. expansum*), MS-PC-01 (*P. chrysogenum*), MS-BC-02 (*B. cinerea*), MS-FO-01 (*F. oxysporum*), MS-AF-05 (*A. flavus*), and MS-AN-06 (*A. niger*). These strains were previously isolated, identified at the species level using standard mycological methods, and preserved under controlled conditions to ensure experimental reliability. All cultures were maintained on potato dextrose agar (PDA; HiMedia, Mumbai, India) slants at 4 °C and subcultured monthly. For assays, conidia were harvested from 7-day-old cultures, suspended in sterile saline (0.85% NaCl; El-Gomhoria Co. (Cairo, Egypt) containing 0.05% Tween 80 (Piochem Laboratory Chemicals (Cairo, Egypt), and adjusted to 2–5 × 10⁴ CFU·mL⁻¹ using the pour plate count technique (Qi et al. 2025).

### Preliminary solvent screening for optimal extraction

Before irradiation experiments, the extraction efficiency of different solvents was evaluated to select the most effective solvent for mushroom bioactives. Dried, non-irradiated *(A) bisporus* powder (5 g) was extracted separately with 50 mL of six solvents of varying polarity (cold water, hot water, methanol, ethanol, and ethyl acetate) for 24 h at room temperature (150 rpm). The extra solvent from the filtrate was evaporated using a rotary evaporator, and reconstituted to 100 mg·mL⁻¹ in the respective solvent. Antifungal activity was assessed against the six test fungi (*P. expansum*, *P. chrysogenum*, *(B) cinerea*, *F. oxysporum*, *A. flavus*, and *A. niger*) using the agar-disc diffusion assay (Akhtar et al. 2024). Based on this screening, one solvent was selected for the subsequent extraction of irradiated mushroom powders.

### Determination of total phenolic content (TPC)

TPC was measured using the Folin–Ciocalteu method (Nurkanto and Julistiono 2014). Briefly, 0.5 mL of extract was mixed with 2.5 mL of 10-fold diluted Folin–Ciocalteu reagent, followed by 2 mL of 7.5% sodium carbonate. After 1 h incubation at room temperature, absorbance was recorded at 765 nm using a UV-Vis spectrophotometer (Jasco V-530, Japan). Gallic acid was used as a standard, and results were expressed as mg gallic acid equivalents (GAE)/g extract.

### DPPH radical scavenging activity

The antioxidant activity of mushroom extracts was assessed using the 1,1-diphenyl-2-picryl-hydrazyl (DPPH) free radical method (Liu et al. 2021). A 0.1 mM DPPH solution in methanol was prepared. One milliliter of DPPH solution was mixed with 1 mL of extract and incubated in the dark for 30 min. Absorbance was read at 517 nm. Tert-butylhydroquinone (TBHQ) was used as the reference antioxidant. Antioxidant activity was calculated using:

$$Antioxidant\;activity\;\left(\%\;inhibition\right)\;=\frac{A\;(control)-A(sample)}{A\;\left(control\right)}\;\times\;100$$

Where:

*A* (control) is the absorbance of the control (DPPH solution without the sample).

*A* (sample) is the absorbance of the test sample (DPPH solution with the mushroom extract).

### Antifungal activity assays and impact of γ-radiation

Antifungal activity was evaluated using the disc diffusion method. PDA plates (9-cm) were seeded with 100 µL of fungal inoculum (2–5 × 10⁴ CFU·mL⁻¹). Sterile paper discs (6 mm, Whatman No. 1) were placed on the agar surface and loaded with 20 µL of extract. Fluconazole discs served as positive controls, and solvent-only discs were used as negative controls. Plates were left for 30 min to allow pre-diffusion, then incubated at 28 °C for 72 h, and inhibition zones were measured in mm (mean ± SD, *n* = 3) (Nurkanto and Julistiono 2014).

### Minimum inhibitory concentration (MIC)

MICs values were determined based on the broth microdilution method (Nurkanto and Julistiono 2014). Briefly, serial dilutions of mushroom extract (2–512 µg·mL⁻¹) were prepared in 96-well plates using Sabouraud broth medium. Wells were inoculated with 2–5 × 10⁴ CFU·mL⁻¹ of conidia. After incubation at 28 °C for 72 h, MIC was defined as the lowest concentration showing no visible growth. Amphotericin B and fluconazole were used as positive controls.

### Mechanistic studies on fungal cells

#### Sorbitol assay for fungal cell wall integrity

Fungal cell wall integrity was assessed using a sorbitol protection assay, as described by Azish et al. (2021). Extracts (2–512 µg/mL) were tested in 96-well plates in media with or without 0.8 M sorbitol. Plates were incubated at 35 °C, and MICs were recorded at 2 and 7 days. Amphotericin B was used as a positive control. All assays were conducted in triplicate.

#### Effect on fungal enzymatic activity

Fungal extracellular enzymes (protease, cellulase, and lipase) were measured spectrophotometrically using standard substrates (casein, carboxymethylcellulose, and p-nitrophenyl palmitate, respectively). Protease activity was determined by mixing 1 mL of cell-free filtrate with 1% casein in 0.2 M sodium phosphate buffer (pH 7.2), then incubating at 37 °C for 30 min. After adding 10% TCA, samples were centrifuged (4,000 rpm, 10 min), and the supernatant’s absorbance was measured at 750 nm using the Lowry method. One unit (U) = 1 µg L-tyrosine released per minute (Ugbede et al. 2023). Cellulase activity was assessed by incubating 0.5 mL of culture supernatant with 0.5 mL of 1% carboxymethyl cellulose (CMC) in 0.05 M sodium acetate buffer (pH 4.8) at 40 °C for 1 h. Reducing sugars were quantified by the dinitrosalicylic acid (DNSA) method. One unit was defined as the amount of enzyme producing 1 µmol of reducing sugar per minute (Ma et al. 2022a, b). Lipase activity was measured by titrating free fatty acids released from an emulsified substrate at pH 7.4 and 37 °C. One unit was defined as the amount of enzyme releasing microequivalents of alkali per mg protein per hour (Geoffry and Achur 2018).

#### Scanning electron microscopy (SEM)

SEM was used to observe morphological changes in *P. expansum*, *B. cinerea*, and *A. niger* treated with extracts at MIC concentrations (de Almeida et al., 2017). After 48 h incubation at 28 ± 2 °C, hyphae from control and treated cultures were fixed in 2.5% glutaraldehyde (4 °C, 2 h), post-fixed in 1% osmium tetroxide, dehydrated with graded ethanol, and dried by critical point drying. Samples were gold-sputtered and observed using a SEM (SEM model ZEISS EVO-15) operated at an acceleration voltage of 30 kV.

#### GC-MS analysis of extract composition

The GC-MS technique was used to identify the components present in the extract (Rafiq et al., 2024). The analysis was performed on an Agilent 7890B GC system coupled with a 5977 A mass selective detector (Agilent Technologies, USA) at the Central Laboratories Network, National Research Centre, Cairo, Egypt. Separation was achieved using an HP-5MS capillary column (30 m × 0.25 mm i.d., 0.25 μm film thickness). Helium was used as the carrier gas at a constant flow rate of 1 mL/min in splitless mode. The injection volume was 1 µL. The oven temperature program was set as follows: initial temperature at 60 °C (held for 2 min), ramped at 5 °C/min to 300 °C, and held for 10 min. Injector and detector temperatures were maintained at 280 °C and 300 °C, respectively. Mass spectra were obtained using electron ionization at 70 eV over a scan range of m/z 50–550. Compound identification was based on comparison of mass spectra with entries in the NIST and Wiley libraries.

### Application of mushroom extract to fresh-cut apples

#### Preparation and treatment of fresh-cut apples

Uniform, defect-free apples (*Malus domestica*) were washed and cut into 2 cm-thick slices. Slices were divided into three groups: (i) control (dipped in sterile distilled water), (ii) treated with 10% (w/v) non-irradiated extract, and (iii) treated with 10% (w/v) irradiated extract (6.0 kGy). All treatments lasted 3 min. Slices were air-dried under aseptic conditions, sealed in polypropylene trays, and stored at 4 ± 2 °C for 10 days.

#### Determination of weight loss and firmness

The rate of weight loss was calculated by measuring the initial and final weights of apple slices during storage using the following equation:

$${Weight}\ {loss} (\%) = \frac{W0 - Wt}{W0} \times 100$$

where W_0_ is the mass of fresh-cut apples before storage (g), and W_t_ is the mass after storage (g). Firmness was assessed using a digital penetrometer (GY-3, Top Instrument Co. Ltd., Zhejiang, China) equipped with a 3.5 mm diameter probe. The probe was inserted to a depth of 1 cm on the peeled surface of each slice, and firmness was recorded as the maximum force (N) during insertion (Luo et al. 2011). Weight loss and firmness measurements were performed every two days during storage (0, 2, 4, 6, 8, and 10 days) at 4 °C.

#### Determination of surface colour and browning index

Surface colour (L*, a*, b*) was measured every 2 days using a chromameter (CR-400, Konica Minolta, Japan) calibrated with a white tile (L* = 97.52, a* = − 5.06, b* = 3.57). Overall colour difference (Δ*E*) was calculated:

$${\Delta}E=\sqrt{\left(\Delta\mathrm{*}\right)2+\left(\Delta\mathrm{a*}\right)2+\left(\Delta\mathrm{b*}\right)2}$$

Where L_0_, a_0_, and b_0_ are the initial colour values at 0 days, while L_1_, a_1_, and b_1_ are the values at storage time.

The browning index was determined as described by Chen et al. (2025a, b). Two grams of apple tissue were homogenized with 5 mL of deionized water, transferred to centrifuge tubes, and centrifuged at 4 °C, 3,000 rpm for 15 min. The absorbance of the supernatant was measured at 420 nm, and the browning index was expressed as A₄₂₀.

#### Microbial analysis of treated apple slices

A 25 g sample of each treatment was homogenized with 225 mL of 0.85% saline using a Stomacher 400 (Seward Ltd., UK). Serial ten-fold dilutions were prepared. Total viable bacteria were counted on Plate Count Agar (PCA; HiMedia, India) incubated at 37 °C for 24–48 h. Yeasts and molds were cultivated on SDA (BioLife, Italy) at 26 ± 2 °C for 5 days. Microbial counts were performed every two days during storage (0, 2, 4, 6, 8, and 10 days) at 4 °C, and the results were expressed as log CFU/g, with measurements done in triplicate (de Chiara et al. 2026).

### Statistical analysis

All data were expressed as mean ± standard deviation (SD) from at least three independent replicates. Microbial counts were log₁₀-transformed before analysis. Statistical analyses were performed using SPSS software (Version 26.0; IBM Corp., Armonk, NY, USA). One-way analysis of variance (ANOVA) was used to assess differences among treatments. When significant differences were detected (*p* < 0.05), means were compared using Duncan’s multiple range test.

## Results and discussion

### Selection of optimal solvent for extraction

Extracts from wild fungi are known for their antimicrobial and antioxidant potential worldwide (Kumar Sharma and Gautam 2017). In this study, six solvents of varying polarity, including cold water, hot water, methanol, ethanol, and ethyl acetate, were evaluated for the extraction of antifungal constituents from *A. bisporus.* Extracts obtained using polar solvents showed stronger antifungal activity than those from less polar solvents **(**Table 1**)**. Ethanol produced the highest inhibition zones against all six fungal strains, with the strongest effect (14 mm inhibition zone) observed against *P. chrysogenum*. The finding is consistent with reports that ethanol effectively extracts phenolic and flavonoid compounds from mushrooms (Das et al. 2024). Diverse solvent polarity provides the extraction of different components, leading to distinct antifungal activities (Naz and Bano 2012). Ethanol was therefore selected for all subsequent extractions following γ-irradiation.

**Table 1:** Antifungal activity of different *A. bisporus* extracts against some fungal strains (zone of inhibition is expressed in mm)

Tested fungal strains	Different solvents					Antifungal agent
	Cold water	Hot water	Methanol	Ethanol	Ethyl acetate	Fluconazole
*P. expansum*	0	9	0	11	0	10
*P. chrysogenum*	0	10	0	14	0	9
*F. oxysporum*	0	9	8	10	8	0
*B. cinerea*	0	10	8	13	0	10
*A. flavus*	0	10	10	10	10	9
*A. niger*	0	9	7	13	0	10

### Effect of γ-irradiation on the extraction bioactivity

#### Total phenolic content

**Fig. 1:**
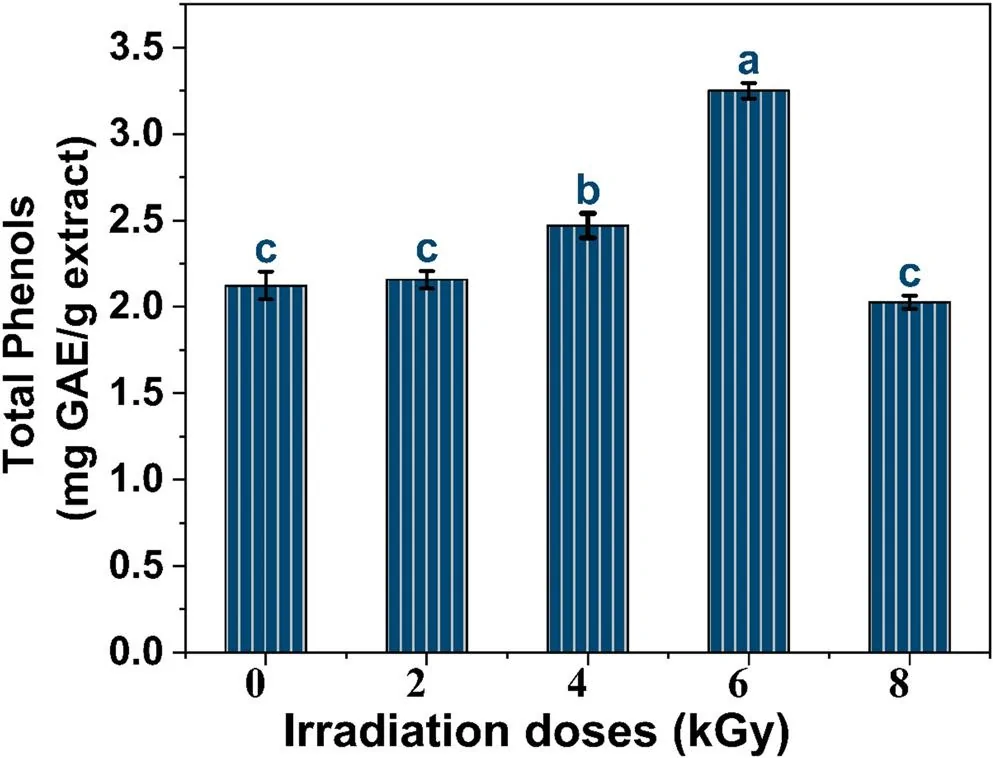
Total phenolic compounds (mg/100g as Gallic acid) of ethanolic extracts from irradiated and non-irradiated *A. bisporus *extract

As shown in Fig. 1, total phenolic content increased significantly with irradiation up to 6.0 kGy, reaching 3.25 ± 1.2 mg GAE/g extract, compared to 2.12 mg GAE/g in the non-irradiated samples (*p* < 0.05). However, a further increase to 8.0 kGy reduced phenolic levels to 2.03 mg GAE/g, likely due to oxidative degradation of sensitive phenolic structures (Amiri et al. 2023). The results exhibited significant differences (*p* < 0.05) between control and irradiated samples at doses of 4.0 and 6.0 kGy, while it was insignificant differences in irradiated samples at dose levels 2.0 kGy. The initial increase in TPC suggests that moderate irradiation enhances phenolic release or formation. However, at higher doses, phenolic degradation likely occurs, as irradiation can cause oxidative breakdown of phenolic structures. These findings align with prior studies showing an optimal irradiation threshold for maximizing bioactive content without degradation in royal jelly, as revealed by Aleksieva et al. (2025), and in edible *Citri Sarcodactylis Fructus* (Wang et al. 2023). These results highlight 6.0 kGy as the optimal dose to enhance phenolic release while avoiding degradation.

#### DPPH radical scavenging activity

The antioxidant activity of phenolics is due to their high tendency to chelate metals. Phenols are very important constituents because of their scavenging ability on free radicals due to their hydroxyl groups (Das et al. 2024). The antioxidant capacity, as assessed by DPPH inhibition, showed a similar trend to TPC (Table 2). Extracts irradiated at 6.0 kGy achieved 46.3% inhibition at 100 µg/mL, compared to 40.9% in non-irradiated extracts and 96.8% for TBHQ. A dose-dependent increase in activity was noted up to 6.0 kGy, followed by a decline at 8.0 kGy. The results show that γ-irradiation can increase the antioxidant capacity of *A. bisporus* extract, but the effect is dose-dependent, peaking at 6.0 kGy and then decreasing with more radiation. Similar results were obtained in *Boletus griseipureus* treated with irradiation doses ranging from 0 to 10 kGy (Chookaew et al. 2019).

**Table 2:** Scavenging activity (%) on DPPH radical of ethanolic extracts from non-irradiated and irradiated dried *A. bisporus* extract

Treatment	Concentration of samples (µg/ml)		
	25	50	100
TBHQ	75.33 ± 2.0	85.3 ± 1.55	96.83 ± 1.6
Control	19.63 ± 1.76	25.5 ± 2.01	40.97 ± 1.4
2.0 kGy	20.30 ± 0.73	30.53 ± 1.2	38.95 ± 1.22
4.0 kGy	22.1 ± 0.24	35.37 ± 1.51	33.47 ± 1.29
6.0 kGy	29.2 ± 0.66	39.77 ± 0.73	46.27 ± 0.38
8.0 kGy	18.7 ± 1.33	23.24 ± 1.47	33.67 ± 1.11

*TBHQ* Tert-Butylhydroquinone

Additionally, several studies have reported similar enhancement of antioxidant activity in other mushroom species following irradiation. For instance, *Lactarius deliciosus* irradiated at 1 kGy showed a significant increase in antioxidant capacity (Fernandes et al. 2017), while *Boletus edulis* treated at higher doses (2, 6, and 10 kGy) also exhibited elevated levels of phenolic compounds and radical-scavenging activity (Fernandes et al. 2013). Furthermore, the improvement in antioxidant capacity following γ-irradiation appears to be dose-dependent. The enhancement observed in *A. bisporus* peaked at 6.0 kGy, after which higher doses reduced activity, a pattern consistent with previous studies on other plant and fungal materials (Shao et al. 2013). Radiation can either enhance or reduce antioxidant activity depending on the dosage, exposure period, and matrix composition (Fernandes et al. 2017; Mladenova et al. 2024). The activation of important biosynthetic enzymes involved in phenolic metabolism, such as phenylalanine ammonia-lyase and peroxidase, has been linked to the positive impact of antioxidant activity enhancement at moderate irradiation doses (Saeid et al. 2024). Similarly, γ-irradiation of *Ziziphus mauritiana* leaves enhanced the accumulation of phytochemicals and improved free radical–scavenging activity up to 12.5 kGy (Khattak and Rahman 2016). On the other hand, Huang and Mau (2006) found that up to 20 kGy of irradiation had no significant effect on the levels of total antioxidant components in *A. blazei*. These findings suggest that low-to-moderate γ-irradiation can enhance the bioactive properties of edible mushrooms by altering their phenolic and sterol profiles.

### Effect of γ-irradiation on the antifungal activity of *A. bisporus*

#### In vitro antifungal activity

**Fig. 2:**
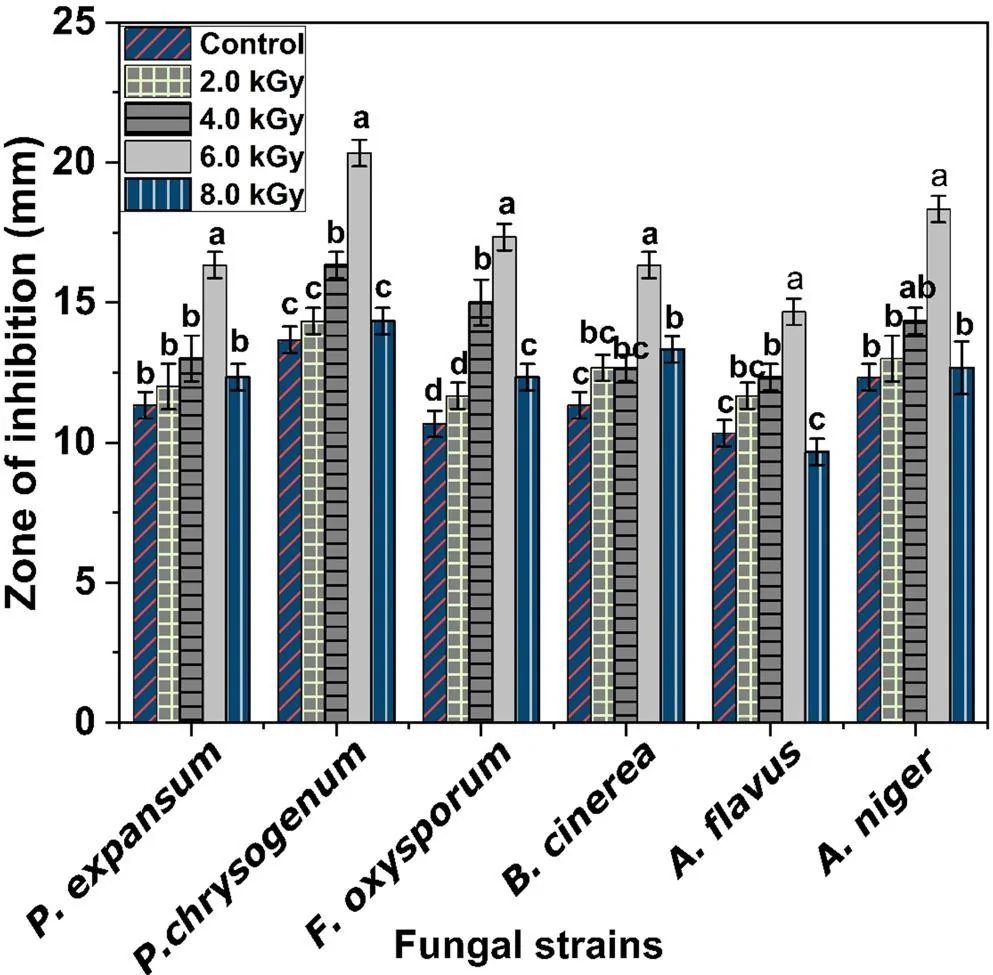
Effect of different doses of γ-irradiation on the antifungal activity of ethanolic extract of* A. bisporus*

To assess the impact of the irradiation on antifungal activity, ethanolic extracts prepared from *(A) bisporus* irradiated at different doses (0, 2.0, 4.0, 6.0, and 8.0 kGy) were evaluated against six major postharvest fungi—*P. expansum*, *P. chrysogenum*, *(B) cinerea*, *F. oxysporum*, *A. flavus*, and *(A) niger*—using the agar-well diffusion assay. The results in Fig. 2 demonstrate that all extracts showed broad-spectrum antifungal activity, with inhibition zones increasing progressively with irradiation dose up to 6.0 kGy (*p* < 0.05). Beyond this dose, activity consistently declined. One-way ANOVA confirmed that irradiation dose significantly affected inhibition zone diameter for all tested fungi (*p* < 0.001; *n* = 3 per dose). The inhibition zone for *P. expansum* increased from 11.3 ± 0.6 mm (control) to 16.3 ± 0.6 mm at 6.0 kGy, a 44% increase (*p* < 0.001). Similarly, at 6.0 kGy, *P. chrysogenum* showed the greatest response, reaching a maximum zone of 20.3 ± 0.6 mm, about 49% larger than the control (13.7 ± 0.6 mm; *p* < 0.001). At 6.0 kGy, inhibition in *F. oxysporum* gradually increased from 10.7 ± 0.6 mm (control) to 17.3 ± 0.6 mm, then declined at 8.0 kGy (12.7 ± 0.6 mm; *p* < 0.001). *(B) cinerea* exhibited a similar pattern, with the zone diameter peaking at 16.3 ± 0.6 mm at 6.0 kGy and decreasing afterward (*p* < 0.001). Regarding the *Aspergillus* species, *A. flavus* showed increased inhibition up to 6.0 kGy (14.7 ± 0.6 mm) but decreased at 8.0 kGy (9.7 ± 0.6 mm; *p* < 0.001), while *A. niger* reached its maximum zone (18.3 ± 0.6 mm) at 6.0 kGy and then declined at 8.0 kGy (12.7 ± 1.2 mm), close to control levels (12.3 ± 0.6 mm).

This suggests that γ-irradiation at suitable levels enhances the extract’s antifungal efficacy by inducing structural or compositional changes in its bioactive components. The findings are comparable with the results of Showkat et al. (2025), who found that γ-irradiation up to 10 kGy improves the bioactivities of Cucurbitaceae seeds against *R. solani* and *C. falcatum*. Exposure to moderate radiation levels (4.0–6.0 kGy) increased the antifungal activity against most fungi, particularly *P. chrysogenum* and *F. oxysporum*, but overexposure (8.0 kGy) reduced biological activity, most likely owing to degradation of key metabolites. The observed pattern aligns with previous reports indicating that moderate irradiation increases extractable phenolics and antioxidants, thereby enhancing antimicrobial efficacy, whereas excessive irradiation leads to structural degradation and loss of activity (Amiri et al. 2023; Fernandes et al. 2013; Kim et al. 2009). Such dose-dependent modulation of bioactivity reflects the balance between beneficial depolymerization and detrimental over-oxidation of phenolic and other bioactive molecules. Mechanistically, moderate γ-irradiation may promote the breakdown of cell walls in mushroom tissue, facilitating the release of bound phenolics, peptides, and secondary metabolites with antimicrobial functions (Kim et al. 2009; Shao et al. 2013).

At higher doses, however, radiation-induced oxidation and polymerization can degrade or inactivate these compounds, explaining the diminished activity observed at 8.0 kGy. The interspecific variation among fungal pathogens likely reflects differences in cell wall composition and membrane susceptibility to the irradiated extract. Notably, *Penicillium* species were among the most responsive, while *Aspergillus* strains showed more moderate inhibition. Therefore, extracts from mushrooms irradiated at 6.0 kGy demonstrated the greatest potential for postharvest protection against common fruit pathogens such as *P. expansum* and *B. cinerea*. These findings support the feasibility of using irradiation-enhanced mushroom extracts as natural antifungal agents.

### Mechanistic insights into antifungal action

#### Sorbitol assay for fungal cell wall integrity

Table 3 shows the MIC values of *A. bisporus* extract in the presence and absence of 0.8 M sorbitol osmotic protector. Sorbitol significantly protected the cells from the inhibitory effects of the mushroom extract, as evidenced by changes in MIC values. MIC values of the irradiated *A. bisporus* increased by two-fold or more in the presence of 0.8 M sorbitol, confirming its interference with fungal cell wall integrity. For instance, MIC for *F. oxysporum* and *P. expansum* increased with sorbitol (125 to 200 mg. mL^− 1^), indicating that cell wall damage is a key antifungal mechanism. Sorbitol acts as an osmotic stabilizer, compensating for weakened cell walls; therefore, a significant MIC shift indicates a cell wall–active mechanism. This observation is consistent with reports that phenolic compounds and sterols can inhibit *β*-1,3-glucan synthase or disrupt chitin assembly (Liu et al. 2024). In this way, wall-targeting antifungal drugs behave similarly (Azish et al. 2021).

**Table 3:** MIC values (µg/mL) of irradiated *A. bisporus* in the absence and presence of sorbitol (0.8 M)

Tested fungal strains		MIC – without sorbitol (µg/mL)		MIC – with sorbitol (µg/mL)	
*P. expansum*		125		200	
*P.chrysogenum*		125		200	
*F. oxysporum*		125		200	
*B. cinerea*		100		175	
*A. flavas*		100		150	
*A.niger*		175		250	

#### Inhibition of fungal enzymatic activity

**Fig. 3:**
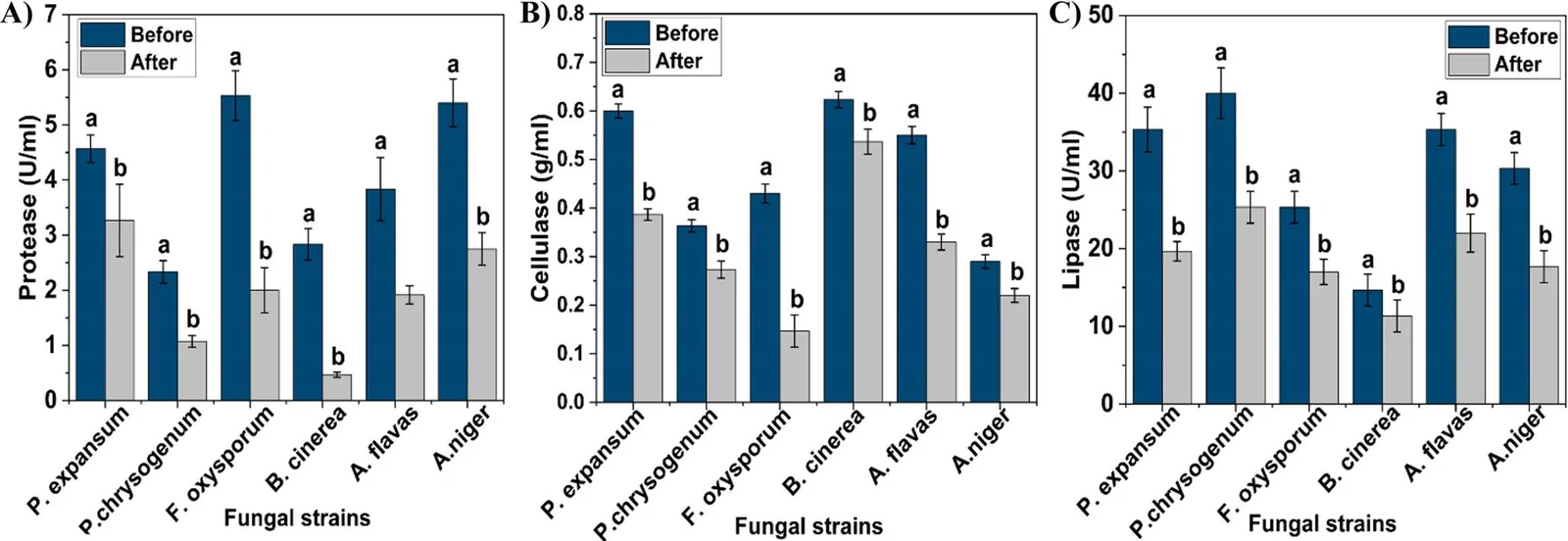
Detection of enzyme activities (**A** for protease, **B** for cellulase, and **C** for lipase enzyme) of the tested fungal strains before and after irradiation of* A. bisporus* extract

Fungal enzymes act as invasive agents that allow pathogens to penetrate host tissues, leading to their deterioration. They can also provide information about mycotoxin release, depending on their physiological state, growth conditions, and environmental factors. The 6.0 kGy extract significantly decreased fungal extracellular enzyme activity, including protease (⁓40%), cellulase (⁓35%), and lipase (⁓45%), which are important for host tissue breakdown (Fig. 3). Reduced enzyme activity can be attributed to either direct inhibition by mushroom extract phenolic chemicals or altered fungal metabolism as a result of membrane and wall damage. Their inhibition suggests that the extract not only kills fungi but also reduces virulence, which is crucial for fresh produce preservation (Añibarro-Ortega et al. 2024). Similar results were obtained in *Aspergillus* species treated with phenolic-rich plant extracts, where protease inhibition was associated with decreased pathogenicity (Min et al. 2025). Collectively, the sorbitol protection and enzyme inhibition data indicate that the antifungal mechanism of the mushroom extract is multifactorial, involving both structural and metabolic disruption.

#### SEM observations

**Fig. 4:**
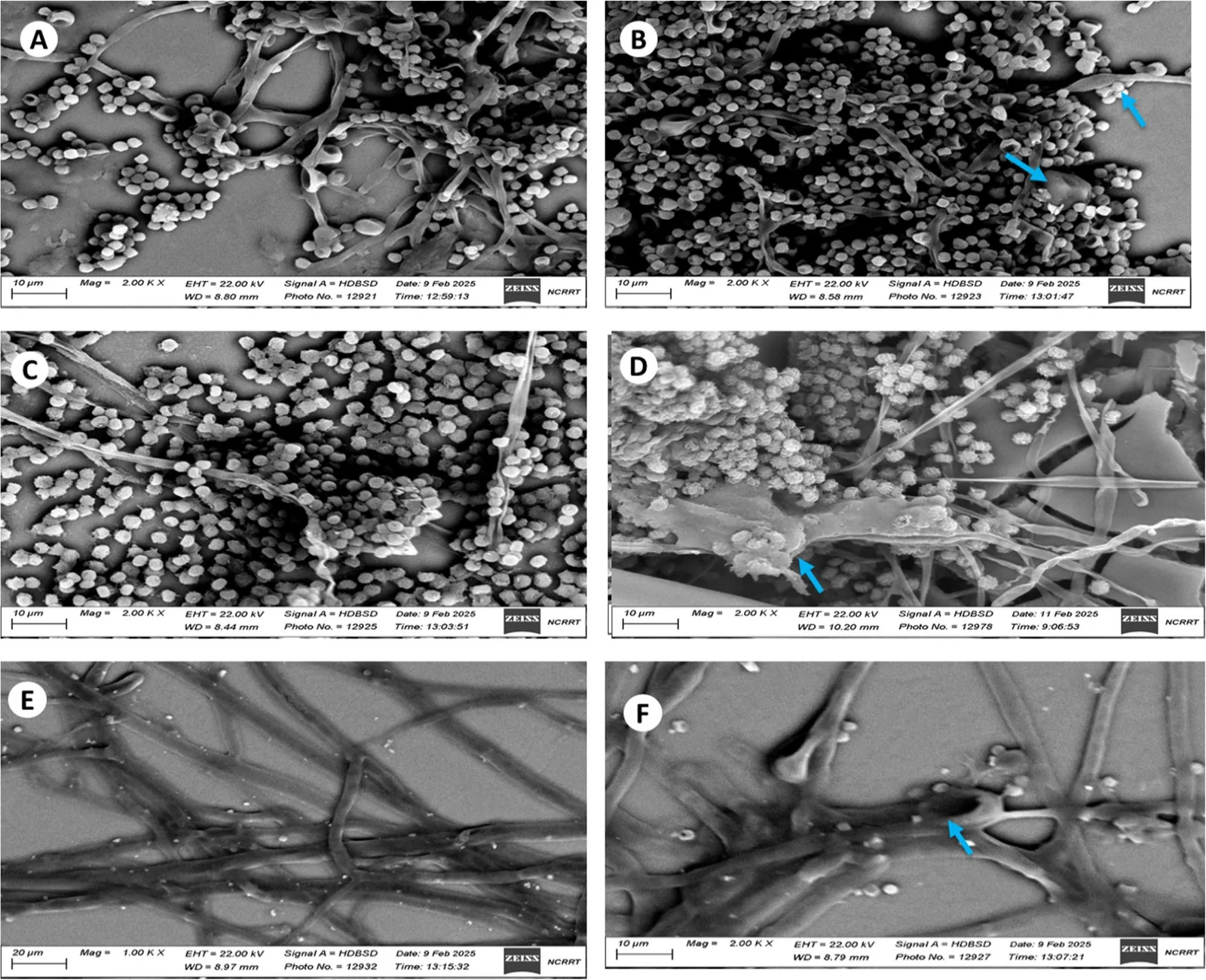
SEM micrographs of *P. expansum*, *B. cinerea*, and *A. niger* non-treated (**A**, **C**, and **E**) and treated with irradiated *A. bisporus* extract (**B**, **D**, and **F**). Arrows indicate surface damage, hyphal collapse, and structural deformation

SEM micrographs provided visual evidence supporting the biochemical assays (Fig. 4). Untreated fungal hyphae exhibited smooth, turgid surfaces with regular branching. In contrast, hyphae exposed to the 6.0 kGy extract showed collapsed hyphae, surface shrinkage, and spore deformation. The effects were more pronounced in *B. cinerea* and *P. expansum*, suggesting a stronger sensitivity. Similar findings were reported by Min et al. (2025), who demonstrated structural disintegration in the hyphae and spores of *M. fructicola*. These morphological changes are likely due to the action of phenolic compounds present in *A. bisporus*, which have been shown to disrupt fungal cell walls and membranes, thereby increasing permeability and leading to cell death. Additionally, the antioxidant properties of these phenolics may contribute to the antifungal activity by generating oxidative stress within the fungal cells (Khan et al. 2016; Liu et al. 2024; Petrova et al. 2007). The SEM findings thus validate the sorbitol and enzyme inhibition results, indicating that γ-irradiation promotes the production of structurally active chemicals that target fungal cell envelopes.

#### GC–MS profiling of bioactive compounds

GC–MS profiling identified multiple compounds whose relative abundance changed in response to γ-irradiation (Table 4). Major constituents included linoleic acid, 9,12-octadecadienoic acid, ergosterol, and neoergosterol derivatives. At 6.0 kGy, the extract showed increased proportions of sterols and medium-chain fatty acids compared to the non-irradiated control. For instance, ergosterol levels increased from 1.03% to 3.09% following irradiation. Additionally, certain compounds, such as neoergosterol and estra-1,3,5(10)-trien-17*β*-ol, were detected only after irradiation. The elevation of ergosterol and neoergosterol suggests oxidative transformation and rearrangement of sterol backbones, processes known to enhance membrane reactivity and antimicrobial potential. Ergosterol, in particular, may enhance antifungal potency by destabilizing fungal membranes (Petrova et al. 2007). Previous studies have demonstrated that irradiation can induce both degradation and formation of secondary metabolites, depending on the absorbed dose (Amiri et al. 2023; Gajbar et al. 2022). On the other hand, the reduction in linoleic acid (9,12-octadecadienoic acid) indicates partial oxidation, which is offset by the rise in antioxidant compounds, contributing to a balanced bioactive profile that supports microbial inhibition and oxidative stability.

**Table 4:** GC–MS profiling of *A. bisporus* extract before and after γ-irradiation (6.0 kGy): Identification and relative abundance of bioactive compounds

Peak	RT (min)	Compound name	Molecular formula	Area Sum %	
				Before	After
1	11.291	3-[N’-(3 H-Indol-3-ylmethylene)-hydrazino]-5-methyl-[1,2,4]triazol-4-ylamine	C_12_H_13_N_7_	1.6	1.03
2	12.453	Dianhydromannitol	C_6_H_10_O_4_	0.78	0.84
3	17.254	DL-Proline, 5-oxo-, methyl ester	C_6_H_9_NO_3_	2.62	2.92
5	25.895	Tetradecanoic acid	C_14_H_28_O_2_	0.2	0.22
6	27.96	Pentadecanoic acid	C_15_H_30_O_2_	0.85	1.15
7	29.122	Hexadecanoic acid, methyl ester	C_17_H_34_O_2_	1.56	1.16
8	30.049	n-Hexadecanoic acid	C_16_H_32_O_2_	4.94	6.56
9	32.297	9,12-Octadecadienoic acid (Z, Z)-, methyl ester	C_19_H_34_O_2_	2.66	2.96
10	32.406	9-Octadecenoic acid (Z)-, methyl ester	C_19_H_36_O_2_	1.12	0.6
11	32.875	Heptadecanoic acid, 16-methyl-, methyl ester	C_19_H_38_O_2_	0.27	0.21
12	33.717	9,12-Octadecadienoic acid (Z, Z)-	C_18_H_32_O_2_	70.41	61.3
14	37.505	Hexanedioic acid, dioctyl ester	C_22_H_42_O_4_	1.22	ND
15	37.733	Isopropyl linoleate	C_21_H_38_O_2_	0.25	ND
16	39.227	Hexadecanoic acid, 1-(hydroxymethyl)-1,2-ethanediyl ester	C_35_H_68_O_5_	1.38	1.93
17	41.91	9,12-Octadecadienoic acid (Z, Z)-, 2,3-dihydroxypropyl ester	C_21_H_38_O_4_	4.41	7.64
19	45.653	9(11)-Dehydroergosteryl benzoate	C_35_H_46_O_2_	1.89	2.41
20	48.251	9(11)-Dehydroergosterol tosylate	C_35_H_48_O_3_S	1.05	1.39
21	48.72	Ergosterol	C_28_H_44_O	1.03	3.09
22	49.08	Neoergosterol	C_27_H_40_O	ND	0.79
23	31.393	Estra-1,3,5(10)-trien-17*β*-ol	C_18_H_24_O	ND	0.29
24	11.898	3-Cyclopentylpropionic acid, cyclopentyl ester	C_13_H_22_O_2_	ND	0.31

*RT* Retention time, *ND*  Not detected

Before = Unirradiated extract; After = extract irradiated at 6.0 kGy

### Preservative effect of irradiated *A. bisporus* extract on fresh-cut apples

#### Weight loss and firmness

**Fig. 5:**
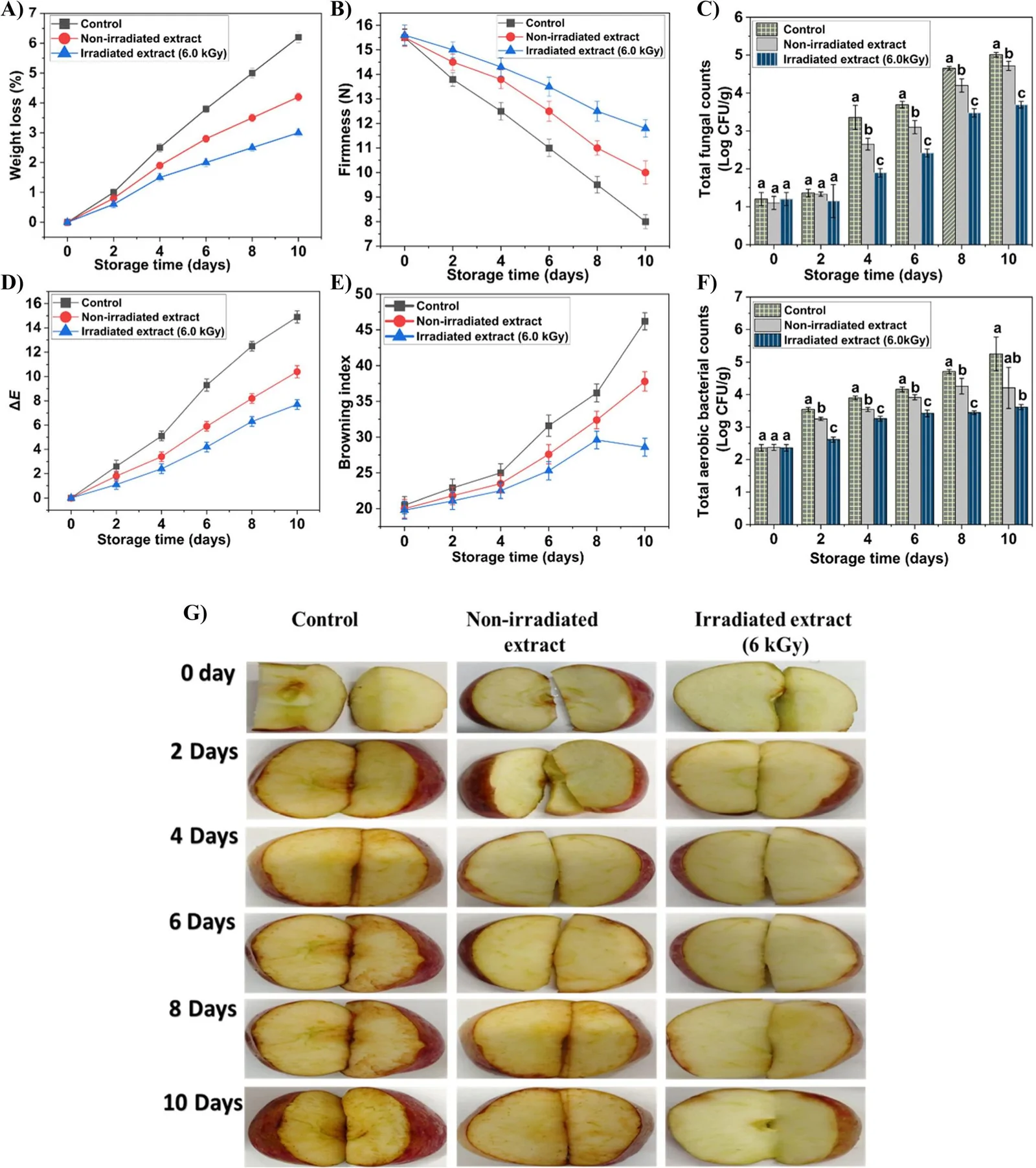
Preservative effects of mushroom extracts on fresh-cut apples during 10 days of storage: (**A**) weight loss rate, (**B**) firmness, (**C**) Total mould and yeast counts, (**D**) color change, (**E**) browning index, and (**F**) Total bacterial counts, and (**G**) visual appearance (shape and colour)

Weight loss in fresh-cut apples results primarily from vapor pressure gradients between the fruit tissue and the surrounding environment. As shown in Fig. 5A, weight loss increased over time in all samples, but was significantly lower in those treated with the irradiated extract (*p* < 0.05). After 10 days, control slices lost 6.2 ± 0.18%, whereas samples treated with the non-irradiated and 6.0 kGy extracts lost 4.2 ± 0.14% and 3.0 ± 0.13%, respectively. The lower weight loss in the irradiated-extract group demonstrates improved moisture retention, likely due to a thin protective layer limiting water evaporation and oxidative membrane damage (Chen et al. 2025a, b; Gong et al. 2023). Fruit firmness, expressed as shearing force, also declined significantly during storage (*p* < 0.05). Control slices decreased from 15.5 N initially to 10.0 N after 10 days, whereas the irradiated-extract group maintained 12.5 N—about 25% higher than controls (Fig. 5B). The retention of firmness is attributed to antioxidant-mediated stabilization of cell walls and inhibition of cell-wall-degrading enzymes such as polygalacturonase, as documented in previous studies (Allegra et al. 2024).

#### Surface colour and browning index

Browning of freshly cut apples arises from enzymatic oxidation of phenolic substrates by polyphenol oxidase (PPO) and peroxidase released during cutting. The irradiated-extract treatment significantly mitigated colour deterioration throughout storage (*p* < 0.05), maintaining lower ΔE and browning index values than both control and non-irradiated treatments (Fig. 5D, 5E). Browning is usually initiated by the enzymatic oxidation of monophenols into ο-diphenols and ο-diphenols into quinones, which undergo further non-enzymatic polymerisation leading to the formation of pigments (Fan 2023). The superior colour preservation corresponds to the higher antioxidant capacity of the irradiated extract, which suppresses PPO-mediated oxidation by scavenging reactive oxygen species and chelating copper ions at the enzyme’s active site (Chen et al. 2025a, b; Fan 2023). Treated slices retained a light, uniform appearance without off-odours, demonstrating the feasibility of using the ethanolic extract from *A. bisporus* irradiated at 6.0 kGy as a natural preservative coating for minimally processed fruits. The minimal differences in color observed between treated and control apple slices indicate that the extract, including after γ-irradiation, did not cause noticeable visual alterations to the fruit. While sensory evaluation of flavor was not conducted in the present study, no visible signs of tissue damage, discoloration, or other quality deterioration were observed during the storage period. This limitation should be addressed in future studies, which could include detailed sensory and physicochemical analyses to comprehensively assess the effect of the treatment on apple quality.

### Microbial load during storage

Treating fresh-cut apple slices with the ethanolic extract of *Agaricus bisporus* irradiated at 6.0 kGy significantly suppressed microbial growth during refrigerated storage (Fig. 5C, 5 F). After 10 days at 4 °C, samples coated with the irradiated extract showed notably lower total microbial counts than the untreated control (*p* < 0.05). Based on log₁₀ CFU·g⁻¹ values, fungal populations decreased by about 1.3 logs, and bacterial populations by 1.6 logs relative to the control. This notable reduction confirms that γ-irradiation improved the antimicrobial effectiveness of the extract, likely through the formation of phenolic derivatives capable of disrupting microbial cell membranes, as previously reported for irradiated phenolic compounds (Liu et al. 2021; Ma et al. 2022a, b). Overall, the irradiated extract demonstrated better control of spoilage microorganisms and helped enhance the microbial stability of fresh-cut apples during cold storage.

Although the antifungal activity of the irradiated *A. bisporus* extract was clearly demonstrated through in vitro assays against major postharvest fungal pathogens, the apple storage experiment in the present study primarily evaluated microbial quality under natural contamination conditions. Further investigation using controlled challenge tests, involving artificial inoculation of apples with specific spoilage fungi followed by treatment with the extract, would provide deeper insight into its protective efficacy directly on fruit tissues. Such experiments are currently underway in our laboratory as part of a related research project, which also includes evaluating the potential effect of the treatment on mycotoxin production.

## Conclusion

This study presents, for the first time, an innovative approach to enhancing the shelf life of fresh-cut apples using gamma-irradiated *A. bisporus* extract as a natural antifungal and antioxidant preservative. Moderate irradiation at 6.0 kGy significantly increased total phenolic content and antioxidant capacity, resulting in stronger antifungal activity against major postharvest fungal pathogens of apples. Mechanistic assays—including sorbitol protection, enzyme inhibition, and SEM analysis—confirmed that the irradiated extract targets fungal cell walls and suppresses virulence-related enzymes. GC–MS profiling indicated irradiation-induced modifications in sterols and fatty acids that contribute to improved bioactivity. When applied to fresh-cut apples, the 6.0 kGy extract reduced microbial load, inhibited browning, and preserved firmness during refrigerated storage, confirming its multifunctional preservative action. Overall, these findings demonstrate that moderate γ-irradiation can enhance the antioxidant and antifungal properties of mushroom-derived bioactives, improving their potential as a natural preservative for fresh-cut apples. The combined effects on oxidative stability, microbial quality, and fruit texture highlight the promise of this approach as a clean-label strategy for extending the shelf life of minimally processed fruits. Further studies evaluating controlled pathogen challenge tests and potential effects on mycotoxin production are currently underway to better understand the protective mechanisms and practical applications of this treatment.

## Supplementary Information

Supplementary Material 1.

## Data Availability

All related data and methods are present in this paper. Additional inquiries can be addressed to the corresponding author.
