# Supplementary material for: Enhancing the antifungal and antioxidant activity of Agaricus bisporus extract using gamma radiation: implications for shelf-life extension of fresh-cut apples
Source: Int Microbiol. 2026 May 2;29(7):1107–20. doi: 10.1007/s10123-026-00806-3 (PMC13653835; doi:10.1007/s10123-026-00806-3)
Supplement: Supplementary file 1 [file 10123_2026_806_MOESM1_ESM.docx]

**Highlights**

- γ-Irradiation of Agaricus bisporus (6.0 kGy) significantly enhanced phenolic content and antioxidant capacity.
- Irradiated ethanolic extract showed broad-spectrum antifungal activity against six apple-spoiling fungi.
- Mechanistic assays (sorbitol protection, enzyme inhibition, SEM) confirmed fungal cell-wall disruption.
- GC–MS profiling revealed irradiation-induced modifications in sterols and fatty acids related to bioactivity.
- Application of the irradiated extract (10 %, w/v) reduced browning, microbial load, and firmness loss in fresh-cut apples during 10 days of storage at 4 °C.
